# Exploring the association between tissue sodium content, heart failure subtypes, and symptom burden: insights from magnetic resonance imaging

**DOI:** 10.3389/fcvm.2025.1458152

**Published:** 2025-01-27

**Authors:** Djawid Hashemi, Karl Jakob Weiß, Patrick Doeblin, Moritz Blum, Radu Tanacli, Hana Camdzic, Hans-Dirk Düngen, Frank Edelmann, Titus Kuehne, Marcus Kelm, Sebastian Kelle

**Affiliations:** ^1^ Deutsches Herzzentrum der Charité, Department of Cardiology, Angiology and Intensive Care Medicine, Berlin, Germany; ^2^ Charité – Universitätsmedizin Berlin, Corporate Member of Freie Universität Berlin and Humboldt-Universität zu Berlin, Berlin, Germany; ^3^ DZHK (German Centre for Cardiovascular Research), Berlin, Germany; ^4^ Berlin Institute of Health at Charité – Universitätsmedizin Berlin, BIH Biomedical Innovation Academy, BIH Charité Digital Clinician Scientist Program, Berlin, Germany; ^5^ Brookdale Department of Geriatrics and Palliative Medicine, Icahn School of Medicine at Mount Sinai, New York, NY, United States; ^6^ Deutsches Herzzentrum der Charité - Institute of Computer-Assisted Cardiovascular Medicine, Berlin, Germany

**Keywords:** heart failure, tissue sodium content, magnetic resonance imaging (MRI), ejection fraction (EF), pathophysiology

## Abstract

**Aims:**

Heart failure (HF) is a complex clinical syndrome with high morbidity and mortality, influenced significantly by sodium balance. Recently, magnetic resonance imaging (MRI) has emerged as a non-invasive method to evaluate tissue sodium load in HF patients. This proof-of-principle study investigates the association between tissue sodium content, assessed by MRI, and HF-related baseline parameters in an outpatient cohort of patients with chronic heart failure, including those with reduced ejection fraction (HFrEF), mildly reduced ejection fraction (HFmrEF), and preserved ejection fraction (HFpEF).

**Methods and results:**

This prospective study included 29 HF patients (10 HFpEF, 12 HFmrEF, and 7 HFrEF) recruited from two centers in Berlin, Germany. Patients underwent MRI to assess tissue sodium content in the lower extremity. Tissue sodium content was analyzed in relation to baseline HF parameters, including renal function, natriuretic peptide levels, clinical signs of congestion, diuretic use, and New York Heart Association (NYHA) functional class. No significant differences in tissue sodium content were observed between the three HF entities. Sodium values did not differ significantly with clinical signs of congestion or diuretic use. No significant correlations were found between tissue sodium content and renal function (eGFR) or natriuretic peptide levels (NT-proBNP) in any HF group overall. However, explorative analyses showed a positive correlation between free (*r* = 0.79, *p* = 0.036) and total (*r* = 0.79, *p* = 0.036) tissue sodium content in the skin and NT-proBNP levels in HFrEF patients, but not in HFmrEF and HFpEF. Similarly, there was a correlation between kidney function and both free (*r* = −0.64, *p* = 0.025) and total (r = −0.61, *p* = 0.035) skin sodium in patients with edema and no prior use of loop diuretics, but no correlation for kidney function and both free and total skin sodium in symptomatic patients with established diuretic therapy or asymptomatic patients with no diuretic therapy.

**Conclusion:**

Our findings provide exploratory insights into the potential diagnostic value of tissue sodium content in HF, particularly in HFrEF patients. With findings showing an association of tissue sodium content with NT-proBNP levels in HFrEF patients and with kidney function in edema patients without prior loop diuretic use, further research is needed to understand the role of tissue sodium content in HF pathophysiology and its potential diagnostic and prognostic implications.

**Trial registration:**

German Clinical Trials Register (DRKS), registration number (DRKS00015615).

## 1 Background

Heart failure (HF) is a major public health issue affecting millions of people worldwide and is associated with high morbidity and mortality (1). Heart failure (HF) is a complex clinical syndrome characterized by the inability of the heart to adequately pump blood, leading to symptoms such as dyspnea, fatigue, and fluid retention. HF can be classified based on left ventricular ejection fraction (LVEF) into heart failure with reduced ejection fraction (HFrEF), mildly reduced ejection fraction (HFmrEF), and preserved ejection fraction (HFpEF). Understanding the underlying pathophysiology and identifying potential biomarkers related to HF is crucial for improving diagnosis, monitoring, and treatment of this debilitating condition.

Magnetic resonance imaging (MRI) has emerged as a powerful tool in cardiovascular imaging, providing detailed and non-invasive assessment of cardiac structure, function, and tissue characterization (2). In the context of HF, MRI can help to identify underlying causes, evaluate ventricular function and remodeling, and guide therapeutic interventions. Moreover, MRI can be employed to assess the sodium content in various tissues, including skin, muscle, and fat, offering a non-invasive method to evaluate tissue sodium load in HF patients (3, 4).

Sodium balance plays a critical role in the development and progression of HF (5, 6). Elevated sodium levels can contribute to fluid retention, increased blood volume, and increased preload, leading to worsening HF (4, 7). Non-invasive and biopsy results suggest interstitial sodium is bound to negatively charged glycosaminoglycans affecting local metabolism and therefore secondary vascular adaptation (8–11). In HF patients, understanding the tissue sodium load may provide valuable insights into the complex interplay between sodium balance and HF progression, potentially leading to the identification of novel therapeutic targets or prognostic markers. Studies exploring the interstitial sodium content in HF patients have been conducted in small patient groups and require further investigation—an analysis with standard-of-care parameters e.g., natriuretic peptide levels, has not been conducted (8).

Considering the potential implications of tissue sodium load in HF pathophysiology, this study aims to conduct an exploratory, proof-of-principle analysis investigating the association between tissue sodium, as assessed by MRI, and HF related baseline parameters in patients with chronic HFrEF, HFmrEF, and HFpEF. By focusing on stable outpatient HF patients, this study provides preliminary insights into tissue sodium content across HF subtypes.

By employing MRI to assess tissue sodium content and investigating its relationship with established HF biomarkers and clinical parameters, this study aims to contribute to the current understanding of HF and inform the development of more personalized and effective management strategies for HF patients.

## 2 Methods

### 2.1 Study design and patient selection

This study was a prospective study conducted at two centers in Berlin, Germany, the Charité—University Medicine Berlin and the German Heart Centre Berlin, between 2017 and 2018. Its rationale and design have been previously described (12–16).

Briefly, subjects were screened for diagnosed HF and an age of at least 45 years. The age criterion of 45 years was chosen to limit age-dependent heterogeneity across the HF subtypes, as the affected age groups for HFrEF, HFmrEF, and HFpEF can differ. This approach aimed to ensure a more homogeneous cohort for analysis while acknowledging that HF patients younger than 45 years may not differ substantially in terms of HF pathophysiology. The initial diagnosis of HF should have been older than 30 days; the patients were required to be in a stable state with no changes in their HF medication and no HF hospitalization within the previous 7 days. Clinical hypervolemia, as applied in this study, is defined as the clinical presentation consistent with an excessive volume of fluid in the intravascular and/or interstitial compartments, including signs such as edema, rales on lung auscultation, and elevated jugular venous pressure. HFrEF was defined as diagnosis of HF, increased N terminal pro brain natriuretic peptide (NT-proBNP) (>220 pg/ml) and LVEF <40%, HFmrEF as the diagnosis of HF, increased NT-proBNP (>220 pg/ml) and 40% ≥ LVEF < 50% as well as HFpEF as diagno-sis of HF, increased NT-proBNP (>220 pg/ml) and LVEF ≥50% at the time of study inclusion. We did not distinguish between the causes for HF for recruiting patients (14).

The study included complied with the Declaration of Helsinki, the protocol was approved by the responsible ethics committees, and all patients gave written informed consent. It was registered at the German Clinical Trials Register (DRKS, registration number: DRKS00015615). The detailed inclusion and exclusion criteria are listed on the webpage of the DRKS.

Additionally, we added the sodium values from studies that assessed the tissue sodium content in healthy controls. These studies were identified via a MEDLINE search for (“sodium” AND “mri” AND “heart failure”) on May 23rd 2023. The MEDLINE search via PubMed was conducted and yielded 90 results. A manual revision was carried out specifically for studies featuring healthy controls with measured sodium values. This process revealed two studies with 9 and 14 healthy control subjects respectively (8, 17). As the values from the literature were only provided as mean and standard deviation values, a direct statistical comparison across groups was not feasible.

### 2.2 ^23^Na MR imaging and quantification of tissue sodium by CMR

As previously described, the CMR images were acquired separately at a 1.5 T (Achieva, Philips Healthcare, Best, The Netherlands) magnetic resonance imaging (MRI) scanner (12–15). Cine images were acquired using a retrospectively gated cine-CMR in cardiac short-axis, vertical long-axis, and horizontal long-axis orientations using a steady-state free precession sequence for volumetry (14, 15).

The additional images for the tissue sodium assessment were acquired using a Philips Ingenia 3.0 Tesla MR scanner (Ingenia R 5.4, Philips Healthcare, Best, The Netherlands)—our approach has previously been described (18–20). In brief, in each study participant, one calf was scanned on the 3.0 Tesla scanner using a ^23^Na send/receive knee coil (Rapid Biomedical, Rimpar, Germany) with a 2D-spoiled gradient echo sequence (18–20). ^1^H-MRI was performed using a fat-saturated inversion-prepared SE sequence.

Selected regions of interest (ROI) were (1) the whole leg, (2) different muscles (triceps surae, peroneus group, grastrocnemius medialis, gastrocnemius lateralis, soleus), (3) tibial bone, (4) skin, and (5) subcutaneous fat.

To distinguish between sodium storage dependent on water, such as in the case of edema, and sodium storage independent of water, for example when sodium is bound to glycosaminoglycans, we paired ^23^Na scans with ^1^H-MRI scans (18, 21). Four calibration vials containing solutes of 10, 20, 30, and 40 mmol/L NaCl were scanned as reference standards, along with the subject's calf, and linear trend analysis was performed to convert ROI intensity to NaCl concentration.

The sodium scan took 35 min out of the full 45-min scan session.

### 2.3 Image analysis

All images were analyzed offline using commercially available software (Medis Suite, version 3.1, Leiden, The Netherlands) in accordance to recent consensus document for quantification of LV function using CMR (22). ^23^Na analysis was performed using Horos (Horos Project, Annapolis, MD, USA).

### 2.4 Endpoints

The endpoint of this analysis was the difference between the HF entities regarding the tissue sodium levels. Further exploration focused on the association of sodium levels with natriuretic peptide as well as renal function parameters (NT-proBNP and estimated glomerular filtration rate, eGFR).

### 2.5 Statistical analysis

Statistical analysis was carried out with R version 3.5.1 (2018-07-02; R Foundation for Statistical Computing, Vienna, Austria) and IBM SPSS Statistics version 29.0.0.0 (241; IBM Corp., Armonk, NY, USA).

Normality of variables was assessed by visual assessment of normality curves and the Shapiro–Wilk test. Given the small sample size, assumptions of normality should be interpreted cautiously. Mean ± standard deviation (SD) was chosen for consistency with prior studies and to facilitate comparison. Spearman's rank correlation was computed to assess correlation for continuous and ordinal data. Kruskal-Wallis tests were specifically used to assess variables. For illustrative purposes linear fitting lines were added when appropriate. Values of *P* < 0.05 were considered statistically significant. Values are reported as mean ± standard deviation, and also as median values enclosed in square brackets.

## 3 Results

### 3.1 Demographic and clinical characteristics

Tissue sodium content was measured in 29 of the 71 study patients—only these patients agreed to the prolonged scanning time aside of the cardiac scanning. The baseline characteristics of the complete study population have been previously reported (13–15, 16).

**Figure 1:**
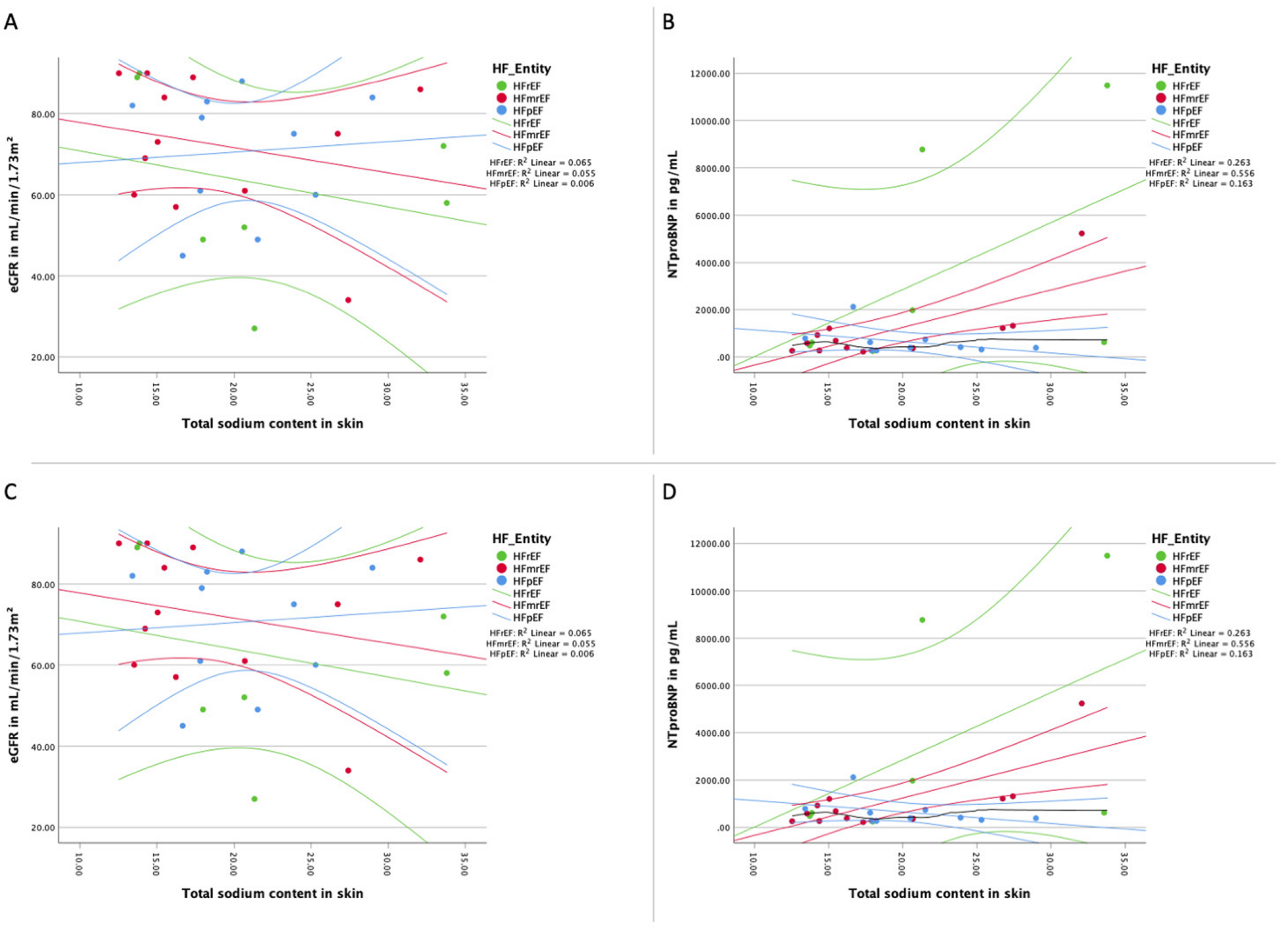
Association of sodium load and natriuretic peptides and renal function. **(A)** Distribution of the total sodium load of the skin of the lower extremity and the renal function for each HF entity with linear regression line for each HF group; **(B)** distribution of the total sodium load of the skin of the lower extremity and natriuretic peptide NT-proBNP for each HF entity with linear regression line for each HF group; **(C)** distribution of the free sodium load of the skin of the lower extremity and the renal function for each HF entity with linear regression line for each HF group; **(D)** distribution of the free sodium load of the skin of the lower extremity and natriuretic peptide NT-proBNP for each HF entity with linear regression line for each HF group. eGFR, estimated glomerular filtration rate measured with creatinine; HF, heart failure;HFpEF indicates heart failure with preserved ejection fraction; HFmrEF, heart failure with mildly reduced ejection fraction; HFrEF, heart failure with reduced ejection fraction; NTproBNP, N-terminal pro brain natriuretic peptide. Linear fitting lines and corresponding *R*^2^ values are for illustrative purposes only.

We successfully scanned 29 patients (11 female, 18 male) included 10 patients presenting with HFpEF, 12 with HFmrEF and seven patients with HFrEF. Table 1 shows the baseline characteristics, HFpEF patients were oldest (78.0 ± 5.9 years), HFmrEF (66.3 ± 10.7 years) patients were youngest, leaving patients with HFrEF (67.7 ± 6.5 years) in between (overall *p*-value = 0.005). The same pattern is true for systolic blood pressure, while both diastolic blood pressure and heart rate were similar across groups. Particularly, the renal function, the natriuretic peptide levels, hematocrit and signs of hypervolemic congestion as well as diuretic use were not different across the HF entities. While the white blood cell count (WBC) was similar across HF entities, we found a significantly altered level of C-reactive protein (CRP) in our patients. Figure 1 presents the association of sodium load with renal function and natriuretic peptide levels in each HF group.

**Table 1:** Patient Characteristics.

	HFpEF (*n* = 10) mean ± SD [median]	HFmrEF (*n* = 12) mean ± SD [median]	HFrEF (*n* = 7) mean ± SD [median]	*P*-value
Age—years	78.0 ± 5.9 [78.9]	66.3 ± 10.7 [68.0]	67.7 ± 6.5 [67.0]	*0*.*005*
Female sex—no. (%)	6 (60)	5 (42)	0 (0)	*0*.*040*
SBP—mmHg	151.4 ± 10.8 [153.0]	131.9 ± 14.2 [134.5]	140.0 ± 8.7 [137.0]	*0*.*003*
DBP—mmHg	83.4 ± 12.7 [88.5]	78.1 ± 7.6 [78.0]	83.3 ± 11.5 [88.0]	0.504
Heart rate—bpm	67.6 ± 8.0 [68.0]	64.7 ± 6.4 [64.5]	65.1 ± 9.6 [67.0]	0.699
BSA—m^2^	1.9 ± 0.2 [1.8]	1.9 ± 0.1 [1.9]	2.0 ± 0.2 [2.1]	0.247
BMI—kg/m^2^	27.4 ± 2.2 [27.1]	26.3 ± 4.7 [25.6]	27.4 ± 3.0 [27.9]	0.413
LVEF—%	60.9 ± 4.7 [61.0]	44.3 ± 3.3 [45.0]	33.9 ± 4.9 [36.0]	*<0*.*001*
LVEDVi—ml/m^2^	69.2 ± 16.6 [65.0]	97.8 ± 13.5 [95.6]	142.9 ± 16.6 [139.0]	*<0*.*001*
Serum potassium—mmol/L	4.2 ± 0.2 [4.2]	4.2 ± 0.5 [4.3]	4.4 ± 0.5 [4.3]	0.678
Serum sodium—mmol/L	140.7 ± 3.1 [141.0]	142.1 ± 1.24 [142.0]	141.1 ± 3.4 [140.0]	0.305
eGFR—ml/min/1.73 m^2^	70.6 ± 15.6 [77.0]	72.3 ± 17.1 [74.0]	62.4 ± 22.8 [58.0]	0.568
Albumin—g/L	41.3 ± 3.1 [41.2]	43.3 ± 2.2 [43.6]	42.2 ± 2.5 [42.4]	
NTproBNP—ng/L	631.1 ± 554.4 [401.5]	1,054.3 ± 1,374.2 ([635])	3,453.1 ± 4,656.6 [(620)]	0.444
Hct(l/L)	0.39 ± 0.03 [0.40]	0.40 ± 0.03 [0.41]	0.43 ± 0.03 [0.41]	0.221
WBC(/nl)	7.79 ± 2.65 [7.77]	7.88 ± 1.29 [8.04]	7.06 ± 1.44 [6.96]	0.658
CRP(mg/L)	3.7 ± 3.2 [2.8]	1,7 ± 1.3 [1.4]	0.8 ± 0.5 [0.6]	0.006
Medication				
Beta-blockers—no. (%) [no—(% on target dose)]	5 (50)	10 (83)	7 (100) [2 (29)]	0.052
ACE-I/ARB/ARNI—no. (%) [no—(% on target dose)]	6 (60)	7 (58)	4 (57) [3 (43)]	0.993
MRA—no. (%) [no—(% on target dose)]	1 (10)	2 (17)	5 (71) [2 (29)]	0.011
Loop diuretic—no. (%)	1 (10)	2 (16.7)	1 (14.3)	0.892
Thiazide/Thiazide-like diuretic—no. (%)	0 (0)	2 (16.7)	0 (0)	0.912
Clinical Hypervolemia	5 (50)	11 (91.7)	3 (42.9)	0.093
Peripheral edema	5 (50)	10 (83.3)	3 (42.9)	0.262
Ascites	0 (0)	0 (0)	1 (14.3)	0.108
Rales	0 (0)	1 (8.3)	1 (14.3)	0.532

All values are given as mean (standard deviation, SD) and [median] or count (percentage). BSA, body surface area (Du Bois for-mula); BMI, body mass index; SBP, systolic blood pressure; DBP, diastolic blood pressure; eGFR, estimated glomerular filtration rate measured with creatinine; HFpEF indicates heart failure with preserved ejection fraction; HFmrEF, heart failure with mildly reduced ejection fraction; HFrEF, heart failure with reduced ejection fraction; LVEDV, left ventricular enddiastolic volume; LVEDVi, LVEDV indexed over BSA; NTproBNP, N-terminal pro brain natriuretic peptide; Hct, hematocrit; WBC white blood cell count; CRP C-reactive protein; ACE-I, angiotensin-converting-enzyme inhibitors; ARB, angiotensin II receptor blockers; ARNI, angiotensin-receptor-neprilysin-inhibitors.'Clinical hypervolemia’ is defined as the clinical presentation consistent with an excessive volume of fluid in the intravascular and/or interstitial compartments, including signs such as edema, rales on lung auscultation, and elevated jugular venous pressure.

**Figure 2:**
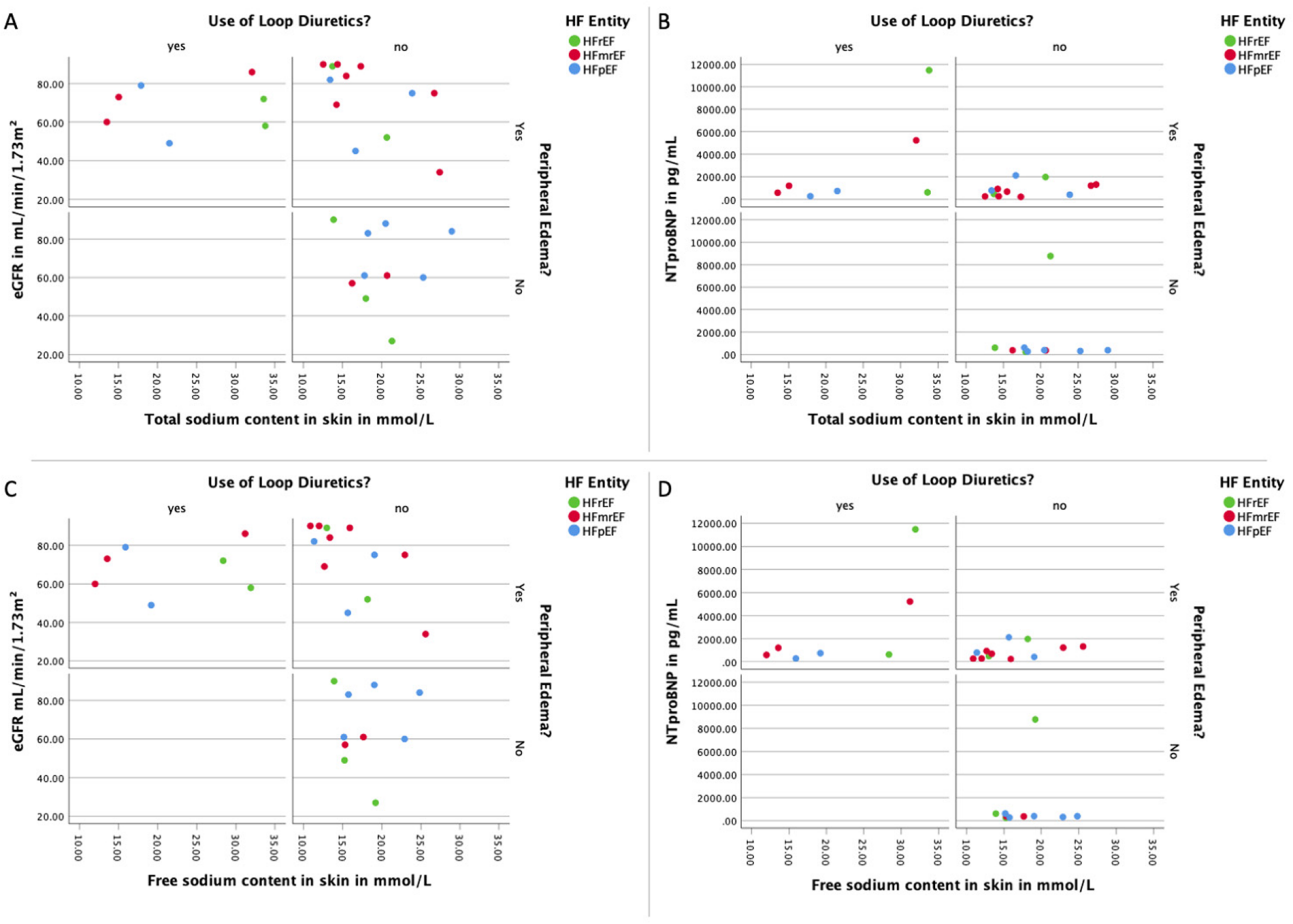
Association of loop diuretics and peripheral edema with sodium load, natriuretic peptides, and renal function. **(A)** Panel with the distribution of the total sodium load of the skin of the lower extremity and the renal function for each HF divided by the use of loop diuretics as well as the presence of peripheral edema; **(B)** panel with the distribution of the total sodium load of the skin of the lower extremity and the natriuretic peptide NT-proBNP for each HF divided by the use of loop diuretics as well as the presence of peripheral edema; **(C)** panel with the distribution of the free sodium load of the skin of the lower extremity and the renal function for each HF divided by the use of loop diuretics as well as the presence of peripheral edema; **(D)** panel with the distribution of the free sodium load of the skin of the lower extremity and the natriuretic peptide NT-proBNP for each HF divided by the use of loop diuretics as well as the presence of peripheral edema. eGFR, estimated glomerular filtration rate measured with creatinine; HF, heart failure; HFpEF indicates heart failure with preserved ejection fraction; HFmrEF, heart failure with mildly reduced ejection fraction; HFrEF, heart failure with reduced ejection fraction; NTproBNP, N-terminal pro brain natriuretic peptide.

Table 2 shows the measured sodium values in different sites across the HF entities. Overall mean tissue sodium content was 22.17 ± 9.03 mmol/L for the whole leg, 24.97 ± 17.57 mmol/L for the skin, and 19.99 ± 5.92 mmol/L for muscle tissue. The sodium values were no different between the HF entities of our studied cohort. The three HF groups were not significantly different with regards to both the tissue sodium as well as the water load. Analyzing the three HF groups separately by the presence of clinical signs of congestion or the use of diuretics did not reveal differences (Figure 2).

**Table 2:** Tissue sodium values.

Sodium values (values in mmol/L)	HFpEF (*n* = 10) mean ± SD [median]	HFmrEF (*n* = 12) mean ± SD [median]	HFrEF (*n* = 7) mean ± SD [median]	*P*-value
Total sodium load				
Triceps surae	20.7 ± 4.9 [19.8]	21.1 ± 8.8 [16.5]	20.5 ± 4.8 [20.9]	0.863
Peroneus group	20.4 ± 4.6 [20.5]	19.0 ± 10.1 [19.0]	17.5 ± 6.9 [19.8]	0.500
Tibial bone	3.1 ± 2.6 [2.9]	3.2 ± 2.4 [3.2]	3.5 ± 4.8 [3.5]	0.766
Gastrocnemius medialis	21.7 ± 5.4 [23.5]	22.6 ± 7.2 [21.3]	23.9 ± 7.2 [20.4]	0.830
Gastrocnemius lateralis	22.4 ± 5.0 [23.6]	22.3 ± 11.6 [15.8]	22.1 ± 6.7 [22.6]	0.820
Soleus	20.2 ± 5.9 [18.7]	20.6 ± 9.3 [16.6]	18.5 ± 3.2 [18.6]	0.779
Whole leg	22.0 ± 5.1 [23.2]	22.1 ± 10.4 [16.0]	28.1 ± 16.7 [24.3]	0.322
Skin	21.0 ± 5.0 [21.0]	20.2 ± 7.4 [16.8]	22.8 ± 9.0 [21.0]	0.555
Subcutaneous fat	22.3 ± 12.9 [17.4]	28.3 ± 20.7 [22.5]	32.4 ± 26.0 [22.5]	0.619
Free sodium load				
Triceps surae	19.2 ± 4.6 [19.2]	19.2 ± 9.0 [14.4]	19.2 ± 5.0 [17.9]	0.741
Peroneus group	18.9 ± 4.4 [18.8]	17.1 ± 9.6 [12.3]	16.3 ± 6.6 [17.5]	0.514
Tibial bone	3.5 ± 3.2 [2.6]	3.7 ± 1.6 [3.7]	3.1 ± 4.3 [1.5]	0.864
Gastrocnemius medialis	20.0 ± 5.0 [22.1]	19.4 ± 8.1 [14.7]	22.1 ± 8.5 [20.0]	0.407
Gastrocnemius lateralis	21.2 ± 5.7 [21.0]	21.2 ± 12.3 [14.5]	20.6 ± 7.0 [20.9]	0.800
Soleus	18.6 ± 5.4 [17.3]	18.6 ± 9.3 [14.6]	17.0 ± 3.1 [16.8]	0.756
Whole leg	20.6 ± 4.7 [21.4]	20.9 ± 10.5 [14.7]	27.4 ± 15.4 [22.5]	0.295
Skin	18.5 ± 4.2 [19.0]	18.4 ± 7.3 [15.6]	20.8 ± 7.7 [18.7]	0.563
Subcutaneous fat	21.0 ± 11.2 [17.3]	27.8 ± 21.6 [20.9]	31.3 ± 28.6 [20.4]	0.602
^1^H-MRI				
Triceps surae	0.4 ± 0.4 [0.4]	0.4 ± 0.1 [0.4]	0.4 ± 0.1 [0.4]	0.809
Peroneus group	0.55 ± 0.1 [0.5]	0.5 ± 0.1 [0.4]	0.4 ± 0.2 [0.4]	0.203
Tibial bone	0.1 ± 0.0 [0.1]	0.1 ± 0.2 [0.1]	0.1 ± 0.1 [0.1]	0.499
Gastrocnemius medialis	0.4 ± 0.0 [0.4]	0.4 ± 0.0 [0.4]	0.4 ± 0.1 [0.4]	0.814
Gastrocnemius lateralis	0.4 ± 0.1 [0.4]	0.4 ± 0.1 [0.4]	0.4 ± 0.0 [0.4]	0.821
Soleus	0.4 ± 0.0 [0.4]	0.4 ± 0.1 [0.4]	0.4 ± 0.1 [0.3]	0.614
Whole leg	0.3 ± 0.1 [0.3]	0.3 ± 0.1 [0.3]	0.3 ± 0.1 [0.3]	0.132
Skin	0.1 ± 0.1 [0.1]	0.1 ± 0.0 [0.0]	0.1 ± 0.1 [0.1]	0.340
Subcutaneous fat	0.1 ± 0.1 [0.1]	0.1 ± 0.1 [0.1]	0.2 ± 0.2 [0.1]	0.382

All values are given as mean (standard deviation, SD) and [median] or count (percentage).

^1^ H-MRI, proton magnetic resonance imaging.

The results of the two identified studies from the literature with values of healthy control subjects, which were not as detailed as our measurement reports, are presented in the Supplementary Material (S1 and S2). Upon evaluation of the values (Supplementary Material S1) and the corresponding error bars (Supplementary Material S2), no significant differences across groups were observed, despite a trend towards lower values in control subjects for muscle sodium measurements. For skin sodium measurements and whole leg values, a significant overlap with measured values from our own cohort was observed.

### 3.2 Correlation with renal function, hematocrit and inflammatory markers

An analysis of the correlation between skin tissue sodium content and renal function, as measured by eGFR, was performed for each HF entity. As shown in Figures 1A, 1C, there was no significant correlation between tissue sodium content (total and free) in the skin of the lower extremity and renal function (eGFR) in any of the HF groups.

Hematocrit, a surrogate marker for the volume status of the patient, was not significantly correlated tissue sodium content (total and free) in the skin of the lower extremity across HF entities. Similarly, we found no significant correlation between WBC or CRP and tissue sodium content (total and free, details are presented in the Supplementary Material S3).

### 3.3 Correlation with natriuretic peptide levels

The relationship between skin tissue sodium content and natriuretic peptide levels (NT-proBNP) was assessed for each HF entity. As depicted in Figures 1B, 1D, a significant positive correlation was found between total and free tissue sodium content in the skin of the lower extremity and NT-proBNP levels in HFrEF patients. There was no significant correlation between tissue sodium content (total and free) in the skin of the lower extremity and NT-proBNP levels in HFpEF and HFmrEF patients (Total sodium content: HFpEF: *r*(8) = −0.47, *p* = 0.174; HFmrEF: *r*(10) = 0.52, *p* = 0.085; HFrEF: *r*(5) = 0.79, *p* = 0.036. Free sodium content: HFpEF: *r*(8) = −0.39, *p* = 0.260; HFmrEF: *r*(10) = 0.57, *p* = 0.055; HFrEF: *r*(5) = 0.79, *p* = 0.036).

### 3.4 Correlation with symptom burden

We further examined the correlation between sodium content in skin tissue and symptomatology, as delineated by clinical manifestations of congestion, the administration of loop diuretics, NT-proBNP concentrations, and renal function. Our data reveals a significant inverse association between renal functionality and both free and total skin sodium in edematous patients absent of prior loop diuretic treatment. However, such correlation was absent in symptomatic patients with an existing diuretic regimen or asymptomatic patients without diuretic therapy. As depicted in Figure 2, no significant discrepancies were discernible in the total and free tissue sodium content of the lower extremity skin when contrasting patients with differing clinical congestion states, loop diuretic usage, and NT-proBNP levels (Total sodium content: signs of congestion and use of loop diuretics: eGFR *r*(5) = −0.14, *p* = 0.760; NTProBNP *r*(5) = 0.57, *p* = 0.180; signs of congestion and no use of loop diuretics: eGFR *r*(11) = −0.61, *p* = 0.035; NTProBNP *r*(11) = 0.40, *p* = 0.199; no signs of congestion and no use of loop diuretics: eGFR *r*(7) = −0.13, *p* = 0.725; NTProBNP *r*(7) = −0.08, *p* = 0.829. Free sodium content: signs of congestion and use of loop diuretics: eGFR *r*(5) = −0.04, *p* = 0.939; NTProBNP *r*(5) = 0.68, *p* = 0.094; signs of congestion and no use of loop diuretics: eGFR *r*(11) = −0.64, *p* = 0.025; NTProBNP *r*(11) = 0.43, *p* = 0.167; no signs of congestion and no use of loop diuretics: eGFR *r*(7) = −0.10, *p* = 0.776; NTProBNP *r*(7) = −0.04, *p* = 0.907).

**Figure 3:**
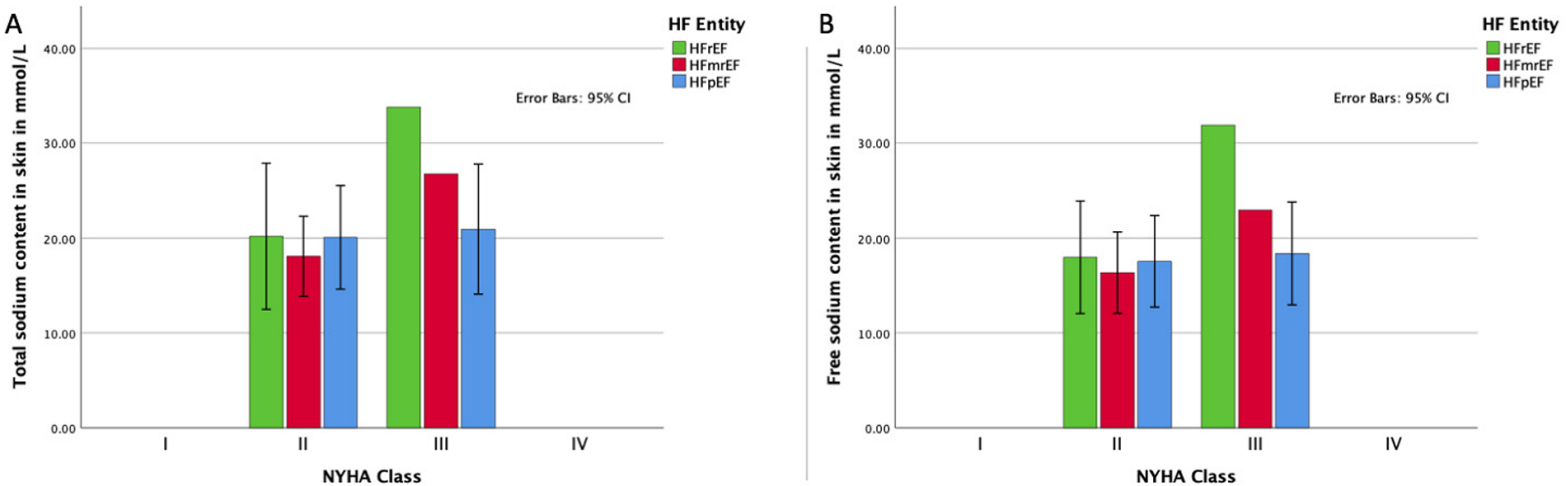
Association of HF functional class and sodium load. **(A)** Distribution of the total sodium load of the skin of the lower extremity for each HF entity in each NYHA functional class with 95% confidence intervals; **(B)** distribution of the free sodium load of the skin of the lower extremity for each HF entity in each NYHA functional class with 95% confidence intervals. HFpEF indicates heart failure with preserved ejection fraction; HFmrEF, heart failure with mildly reduced ejection fraction; HFrEF, heart failure with reduced ejection fraction; NYHA, New York Heart Association.

Furthermore, we assessed the relationship between tissue sodium content and NYHA functional class for each HF entity. Figure 3 presents the distribution of total and free sodium load of the skin of the lower extremity across NYHA functional classes (I-IV) for each HF group. As shown in Figures 3A, 3B, there was no significant difference in the total and free sodium load of the skin of the lower extremity between the different NYHA functional classes for any of the HF entities (Total sodium content: HFpEF: *r*(8) = 0.07, *p* = 0.845; HFmrEF: *r*(10) = 0.31, *p* = 0.334; HFrEF: *r*(5) = 0.61, *p* = 0.144. Free sodium content: HFpEF: *r*(8) = 0.14, *p* = 0.695; HFmrEF: *r*(10) = −0.31, *p* = 0.334; HFrEF: *r*(5) = 0.61, *p* = 0.144), with 95% confidence intervals illustrating overlapping ranges across the classes.

## 4 Discussion

This study represents a proof-of-principle analysis of tissue sodium content in HF patients, including those with HFpEF and HFmrEF, using MRI. Our results discerned a significant correlation between NT-ProBNP levels and both free and total skin sodium content exclusively within the HFrEF group, as well as an inverse correlation between renal function and both free and total skin sodium content in edematous patients without prior diuretic use.

Our preliminary findings may suggest an intriguing role for ^23^Na MRI in determining skin sodium measurements in specific HF patients, thereby facilitating the assessment of volume status and guidance of diuretic up-titration. Notably, our study postulates this novel marker to be independent of both renal function and traditional classifications like NYHA. However, no consistent correlations were found in HFmrEF or HFpEF, indicating that sodium content alone may not differentiate HF subtypes.

The stable, recompensated condition of our cohort is highlighted as the measured values of muscle, skin and whole leg sodium values lies in the same range as the reported values of healthy subjects from the literature as illustrated in S1 and S2.

The pivotal role of sodium balance in the pathophysiology of HF is a well-documented phenomenon (8, 23). Increased sodium levels are implicated in fluid retention, augmented blood volume, and elevated preload, thereby decompensating HF (23). Evidence from non-invasive and biopsy investigations indicates that interstitial sodium is bound to negatively charged glycosaminoglycans, potentially impacting local metabolism and precipitating secondary vascular adaptation (24). Despite this, comprehensive studies probing interstitial sodium content in HF patients are scant and warrant further exploration, especially concerning standard-of-care parameters like natriuretic peptide levels.

Our research enhances current understanding of HF and tissue sodium content by delivering an exploratory analysis of this metric across patients with HFrEF, HFmrEF, and HFpEF. Our findings suggest the absence of significant differences in tissue sodium content among the three HF cohorts, thereby implying that tissue sodium content may not serve as a distinctive biomarker for differentiating HF entities. Contrary to prior research signifying an inverse association between tissue sodium content and renal function in HF patients, our study did not discern any significant correlations (25). The lack of correlation could be attributed to the relatively smaller sample size or the inclusion of stable HF patients, potentially misrepresenting the broader HF populace. Investigations with larger, more diversified patient cohorts are required to comprehend the relationship between tissue sodium content and renal function in HF more effectively.

We observed no significant correlations between tissue sodium content and NT-proBNP levels in HFmrEF and HFpEF patients. Despite our limited numbers, the positive association reported between tissue sodium content and natriuretic peptide levels in previous HFrEF studies cannot be conclusively extrapolated to other HF entities based on our data. The discrepancy between our study and prior research could again be attributed to the limited sample size and the inclusion of chronic HF patients (3, 8, 17, 19, 21, 25). Further exploration in larger, more diversified HF patient cohorts is warranted to understand the association between tissue sodium content and natriuretic peptide levels better.

### 4.1 Limitations

There are several limitations to our study that should be acknowledged. First, the lack of significant differences in tissue sodium content across HF entities may partly reflect in particular the small sample size and the stable outpatient cohort included in this study. These findings suggest that tissue sodium content alone may not sufficiently distinguish between HF subtypes in this population. The small sample size impacts statistical power and the robustness of the results, particularly for confidence intervals shown in Figure 1. Outliers may influence these findings, and future studies with larger cohorts are needed for validation. Second, the inclusion of patients with stable HF may not accurately represent the broader HF population, and further studies with larger and more diverse patient populations are needed to confirm our findings. Diuretic use in the population was determined by the treating physicians and was not controlled in the study design. This variability could influence sodium measurements and should be addressed in future studies. Third, the study was conducted at two centers in Berlin, Germany, which may limit the generalizability of our results to other populations or healthcare settings. Fourth, our use of historical controls from different institutions for comparison introduces an additional limitation. We acknowledge that differences in institutional protocols and patient demographics may introduce variability, limiting direct comparisons. Differences in institutional protocols and patient demographics could affect the validity of these comparisons. Fifth, it's important to note that the use of loop diuretics among study participants was determined by their treating physicians and was not a controlled variable in our study. Finally, our study was an exploratory analysis, and future prospective, longitudinal studies are needed to better understand the relationship between tissue sodium content and HF progression, as well as the potential for tissue sodium content to serve as a prognostic marker or therapeutic target in HF.

## 5 Conclusion

In this exploratory, proof-of-principle study, we investigated tissue sodium content across HF subtypes using MRI. While previous studies have shed light on tissue sodium content in the context of HFrEF, our work adds a new dimension to the existing knowledge by extending this investigation to HFmrEF and HFpEF. This preliminary analysis serves as a stepping stone for future research in this sphere.

Although we found no significant differences in tissue sodium content among the three HF entities and a lack of consistent correlations with renal function and NT-proBNP levels, our data suggest a potential role for tissue sodium measurements in assessing volume status and guiding diuretic therapy in HF patients.

The limited sample size in our study calls for further research with larger and more diverse patient cohorts to elaborate on these initial findings. Larger, multicenter studies are needed to validate these findings, explore their implications, and assess the potential of tissue sodium content as a diagnostic marker in HF.

## Data Availability

The raw data supporting the conclusions of this article will be made available by the authors, without undue reservation.
