# Supplementary material for: Exploring the association between tissue sodium content, heart failure subtypes, and symptom burden: insights from magnetic resonance imaging
Source: Front Cardiovasc Med. 2025 Jan 27;12:1458152. doi: 10.3389/fcvm.2025.1458152 (PMC11807970; doi:10.3389/fcvm.2025.1458152)
Supplement: Supplementary file 1 [file Datasheet1.pdf]

S1. Comparison of sodium values across assessed in HF groups and control patients in literature

	**HFpEF**<br>(n = 10)<br>mean ± SD	**HFmrEF**<br>(n = 12)<br>mean ± SD	**HFrEF**<br>(n = 7)<br>mean ± SD	**Controls Hammon et al. 20158**<br>(n = 9)<br>mean ± SD	**Controls Hammon et al. 201717**<br>(n = 14)<br>mean ± SD
Muscle (Triceps surae in HF patients)	20.7 ± 4.9	21.1 ± 8.8	20.5 ± 4.8	18.3 ±2.5	16.6 ± 2.1
Skin	21.0 ± 5.0	20.2 ± 7.4	22.8 ± 9.0	21.1 ± 2.3	17.9 ± 3.2
Whole leg	22.0 ± 5.1	22.1 ± 10.4	28.1 ± 16.7	18.2 ± 2.5	n.a.
**Image Acquisition Setup Details**					
Total acquisition time (TA; min)	20.5			13.0	13.0
Echo time (TE; ms)	2.14			2.07	2.07
Repetition time (TR; ms)	100			100	100
Flip angle (FA; °)	90			90	90
Averages	196			128	128
Resolution (mm)	3 × 3 × 30			3 × 3 × 30	3 × 3 × 30
Field Strength	3T			3T	3T
Scanner	Ingenia R 5.4			Magnetom Verio	Magnetom Verio
23Na send/receive knee-coil	Rapid Biomedica			Stark-Contrast	Stark-Contrast
No. of centers	Single Center			Single Center	Single Center

n.a.: not available. SD: standard deviation. HFpEF, HFmrEF and HFrEF values were measured in this study. Control subject values (grey background) are values from the literature, Hammon et al. 2015 and Hammon et al. 2017.[8, 17] The measurements of the control subjects were a conducted with a custom-made 23Na knee-coil (Stark Contrast, Erlangen, Germany) at 3.0 Tesla with an MRI scanner (Magnetom Verio, Siemens Health- care, Erlangen, Germany).[8, 17] In the literature data, the values for muscle were assessed at the largest circumference of the leg, there were no further details in which muscles the values were assessed. In the measured values of our study we used triceps surae values for the comparison.
Whole leg sodium measurements were not provided by Hammon et al., 2017.[17]

S2. Illustrative comparison of sodium values across assessed in HF groups and control patients in literature

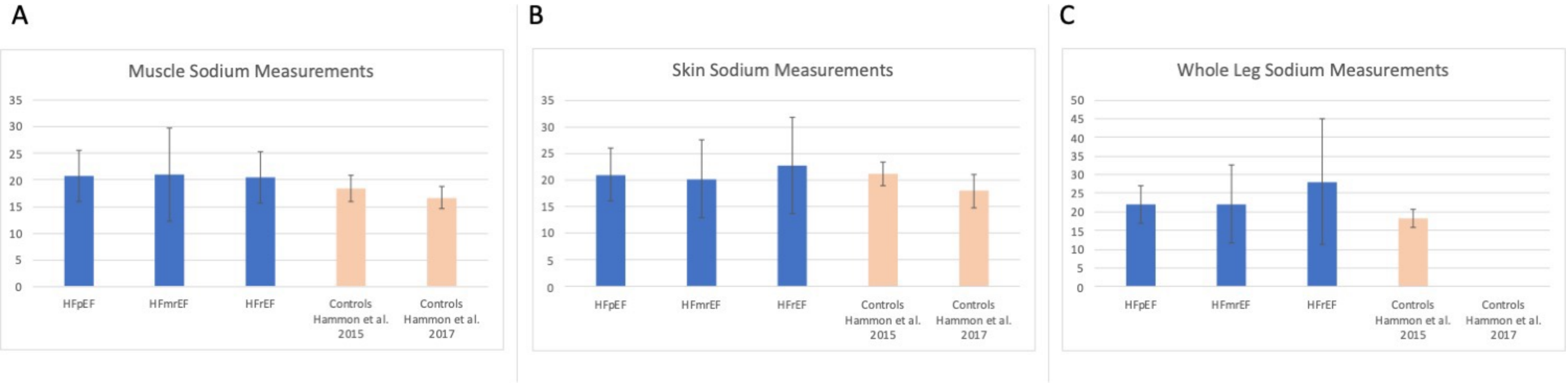

Illustration of the values shown in S1. Blue: Measured values in this study. Rose: Values from the literature, Hammon et al. 2015 and Hammon et al. 2017.[8, 17] Boxes indicate mean values; error bars indicate standard deviation.

A: muscle sodium measurements. In the literature data, the values for muscle were assessed at the largest circumference of the leg, there were no further details in which muscles the values were assessed. In the measured values of our study we used triceps surae values for the comparison.

B: skin sodium measurements.

C: whole leg sodium measurements, no data from the Hammon et al. 2017 provided for this parameter.[17]

S3. Correlation between skin sodium (total and free) and laboratory markers

	HFpEF (n=10)		HFmrEF (n=12)		HFrEF (n=7)	
	Total sodium load (Skin)	Free sodium load (Skin)	Total sodium load (Skin)	Free sodium load (Skin)	Total sodium load (Skin)	Free sodium load (Skin)
Hct (l/l)	r(8) = 0.207 (p = 0.567)	r(8) = 0.255 (p = 0.476)	r(10) = -0.221 (p = 0.491)	r(10) = -0.294 (p = 0.353)	r(5) = -0.234 (p = 0.613)	r(5) = -0.234 (p = 0.613)
WBC (/nl)	r(8) = -0.067 (p = 0.855)	r(8) = 0.079 (p = 0.829)	r(10) = 0.07 (p = 0.829)	r(10) = -0.021 (p = 0.948)	r(5) = 0.107 (p = 0.819)	r(5) = 0.107 (p = 0.819)
CRP (mg/l)	r(8) = 0.091 (p = 0.803)	r(8) = 0.285 (p = 0.425)	r(10) = -0.497 (p = 0.100)	r(10) = -0.431 (p = 0.162)	r(5) = 0.655 (p = 0.111)	r(5) = 0.655 (p = 0.111)

CRP, C-reactive protein; Hct, hematocrit; WBC, white blood cell count
